# Improved Solubility and Stability of a Thermostable Carbonic Anhydrase via Fusion with Marine-Derived Intrinsically Disordered Solubility Enhancers

**DOI:** 10.3390/ijms25021139

**Published:** 2024-01-17

**Authors:** Byung Hoon Jo

**Affiliations:** Division of Life Science, Research Institute of Life Science, and Anti-Aging Bio Cell Factory Regional Leading Research Center (ABC-RLRC), Gyeongsang National University, Jinju 52828, Republic of Korea; jobh@gnu.ac.kr

**Keywords:** solubility enhancer, NEXT tag, intrinsically disordered protein, carbonic anhydrase, *Thermovibrio ammonificans*, CO_2_ capture

## Abstract

Carbonic anhydrase (CA), an enzyme catalyzing the reversible hydration reaction of carbon dioxide (CO_2_), is considered a promising biocatalyst for CO_2_ reduction. The α-CA of *Thermovibrio ammonificans* (taCA) has emerged as a compelling candidate due to its high thermostability, a critical factor for industrial applications. However, the low-level expression and poor in vitro solubility have hampered further utilization of taCA. Recently, these limitations have been addressed through the fusion of the NEXT tag, a marine-derived, intrinsically disordered small peptide that enhances protein expression and solubility. In this study, the solubility and stability of NEXT-taCA were further investigated. When the linker length between the NEXT tag and the taCA was shortened, the expression level decreased without compromising solubility-enhancing performance. A comparison between the NEXT tag and the NT11 tag demonstrated the NEXT tag’s superiority in improving both the expression and solubility of taCA. While the thermostability of taCA was lower than that of the extensively engineered DvCA10, the NEXT-tagged taCA exhibited a 30% improvement in long-term thermostability compared to the untagged taCA, suggesting that enhanced solubility can contribute to enzyme thermostability. Furthermore, the bioprospecting of two intrinsically disordered peptides (Hcr and Hku tags) as novel solubility-enhancing fusion tags was explored, demonstrating their performance in improving the expression and solubility of taCA. These efforts will advance the practical application of taCA and provide tools and insights for enzyme biochemistry and bioengineering.

## 1. Introduction

Carbonic anhydrase (CA, EC 4.2.1.1) is a widespread enzyme that holds great importance for physiological processes such as carbon dioxide (CO_2_) transport, various CO_2_/HCO_3_^−^-related metabolisms, photosynthesis, pH homeostasis, and biocalcification, by catalyzing the reversible hydration of CO_2_: CO_2_ + H_2_O ↔ HCO_3_^−^ + H^+^ [1]. CA enzymes are classified into eight distinct types, among which α-type CAs are the best-known and the most studied [2]. Due to the ultrafast kinetics of CA exhibiting a *k*_cat_ of up to 4.4 × 10^6^ s^−1^ and its proteinaceous nature that can be produced via renewable biological routes, CA has been considered a promising biocatalyst for enzyme-based CO_2_ capture, utilization, and storage (CCUS) technologies [3]. By accelerating CO_2_/HCO_3_^−^ interconversion and thereby overcoming the low CO_2_ solubility in aqueous conditions, CA can improve CO_2_ absorption/desorption [4], mineral carbonation [5], C1-platform chemical synthesis [6,7,8,9], and photoautotrophic microbial growth [10,11,12]. Despite these strengths, two primary challenges limit its practical application: low stability under high-temperature conditions required for efficient CCUS and high enzyme production costs [3,13].

The α-type CA (taCA) of *Thermovibrio ammonificans* type strain HB-1^T^, isolated from a deep-sea hydrothermal vent, has been the most thermostable CA among naturally occurring bacterial CAs known to date, supported by both experimental results and computational simulations [14,15]. Due to its outstanding and promising thermostability, taCA has emerged as a compelling candidate for further enzyme engineering to improve its thermostability [15,16,17]. A large amount of enzymes should be obtained to accelerate protein engineering research and the practical utilization of the enzymes. However, the expression of taCA has been reported to be insufficient in microbial *Escherichia coli* hosts. Moreover, due to its intrinsically poor solubility, taCA is susceptible to aggregation and precipitation under low-salt conditions [14].

The NEXT tag has been employed as a fusion tag to address the challenges associated with both the low-level expression and poor solubility of taCA [18,19]. The NEXT tag, a 53 amino acid-length peptide with a molecular mass of 5.5 kDa, originated from the N-terminal extension sequence of α-type CA (hmCA) in the marine bacterium *Hydrogenovibrio marinus* [18,20]. Despite its small size, the NEXT tag outperforms commercially available fusion tags, such as *E. coli* maltose-binding protein (MBP) and *Schistosoma japonicum* glutathione S-transferase (GST), which have molecular masses of 40 kDa and 26 kDa, respectively. This superior performance is attributed to a distinctive feature of the NEXT tag: it is an intrinsically disordered protein (IDP) that can entropically exclude neighboring macromolecules, thereby preventing protein aggregation [18,21]. However, no comprehensive investigation has been conducted to date regarding the solubility and stability of the NEXT-tagged taCA.

This study examined the length effect of a flexible linker on the expression and solubility of taCA. A comparative analysis was conducted between the NEXT tag and the previously known NT11 tag [22], assessing their influence on the expression and solubility of taCA. Notably, the impact of the NEXT tag on the long-term thermal stability of taCA was explored. Additionally, novel IDP-based solubility-enhancing tags were discovered, and their potential was investigated to enhance the expression and solubility of taCA.

## 2. Results and Discussion

### 2.1. Effects of Length of Flexible Linker

**Figure 1:**
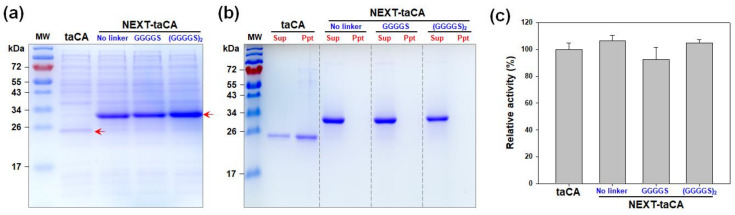
Effect of length of flexible linker on NEXT-taCA. (**a**) Soluble expression level. Protein expression was conducted at 37 °C, and the soluble fraction was analyzed by SDS–PAGE followed by Coomassie blue staining. The arrow indicates the band positions of recombinant proteins. (**b**) In vitro solubility of purified enzyme. After dialysis against 20 mM sodium phosphate buffer (pH 7.5), protein precipitates were separated from soluble supernatants via centrifugation and analyzed by SDS–PAGE. Lanes: MW, molecular mass marker; Sup, supernatant; Ppt, precipitate. (**c**) Activity of purified enzyme. The activities were measured by CO_2_ hydration assay and normalized to the activity of the untagged taCA. Error bars represent standard deviations from two independent experiments.

In the previous study, the NEXT tag was fused to the taCA along with the flexible linker (GGGGS)_×2_ [18]. The fusion of the NEXT tag significantly improved the expression level and the solubility of taCA, minimally affecting the enzymatic properties such as activity and stability [18]. However, the effect of linker length on the expression level, solubility, and activity of NEXT-taCA has not been investigated. As optimization of linker length may enhance protein expression yield [23], two additional fusion proteins featuring distinct linker lengths were subsequently constructed: one where the NEXT tag and taCA were directly attached without a linker and the other where they were fused via a shorter linker (one GGGGS). All variants of NEXT-tagged taCA exhibited high expression levels in soluble forms, whereas, as previously demonstrated, the soluble expression of untagged taCA was considerably lower (Figure 1a). Notably, the original construct with the (GGGGS)_×2_ linker exhibited the highest expression, showing approximately 50% and 37% higher levels than those without a linker and with the shorter linker, respectively.

Despite a protein being expressed in a soluble and folded state, the protein in its isolated state may still form aggregates when possessing low intrinsic, in vitro solubility [24,25]. The taCA enzyme, known for its low in vitro solubility, aggregates under low-salt buffer conditions [18]. The low solubility of taCA appears to be correlated with its positively charged surface [26,27]. To assess the impact of the linker length on the in vitro solubility of NEXT-taCA, the purified proteins were exposed to a low-salt buffer (20 mM sodium phosphate, pH 7.5) without additional supplementation of NaCl, and any resulting protein precipitates were analyzed via SDS–PAGE. As previously demonstrated, the untagged taCA displayed significant precipitates, whereas all the NEXT-tagged taCA enzymes remained entirely soluble, irrespective of the linker length (Figure 1b). This result indicates that the linker length does not influence the solubility of NEXT-taCA. Furthermore, the linker length did not significantly affect the specific enzyme activity (Figure 1c). Consequently, the NEXT-taCA with the (GGGGS)_×2_ linker was selected for all subsequent experiments.

### 2.2. Comparison of NEXT Tag with NT11 Tag

The NT11 tag, consisting of 11 amino acids (VSEPHDYNYEK), is derived from the N-terminal domain of a carbonic anhydrase found in *Dunaliella* species. Due to its small size and notable efficacy, the NT11 tag has gained recognition as a promising tag for the expression of recombinant proteins, including taCA [22]. In this study, the performance of the NT11 tag was assessed and compared with that of the NEXT tag in enhancing protein expression and in vitro solubility of taCA.

**Figure 2:**
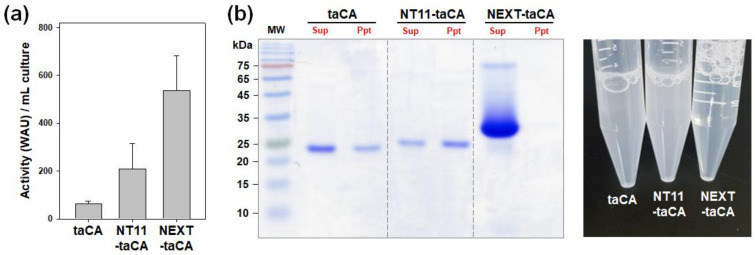
Comparison of NEXT-taCA with NT11-taCA. (**a**) Activity of cell lysate. Error bars represent standard deviations from three independent experiments. (**b**) In vitro solubility of purified enzyme. After dialysis against 20 mM sodium phosphate buffer (pH 7.5), protein precipitates were analyzed by SDS–PAGE followed by Coomassie blue staining (**left**) and by photograph (**right**). Lanes: MW, molecular mass marker; Sup, supernatant; Ppt, precipitate.

First, protein expression levels were roughly assessed by quantifying the enzymatic activities within total cell lysates (Figure 2a) based on the fact that the fusion of either the NT11 tag or the NEXT tag did not result in a statistically significant impact on the enzymatic activity of taCA [14,22]. The CO_2_ hydration activity of the NT11-taCA lysate measured 207.8 WAU mL^−1^, exhibiting a 3.3-fold increase compared to the untagged taCA lysate (63.8 WAU mL^−1^). The activity of the NEXT-taCA lysate (536.2 WAU mL^−1^) was 8.4-fold higher than that of the untagged taCA lysate, which agrees well with the reported value (8-fold) determined through densitometric analysis of band intensities on protein gels [18]. The production yield of NEXT-taCA was estimated to be 104 ± 28 mg L^−1^ by calculation based on the specific activity (5175 WAU mg^−1^) of the purified NEXT-taCA. These findings substantiate that the NEXT tag surpasses the NT11 tag in enhancing the functional expression level of taCA in *E. coli*.

Next, the in vitro solubilities of the tagged proteins were compared by observing protein precipitation following dialysis against a low-salt buffer. Unexpectedly, the NT11-taCA exhibited a significant amount of protein precipitation, rendering the enzyme solution turbid (Figure 2b). The precipitation of NT11-taCA appeared to be more pronounced than that of the untagged taCA. This result suggests that the NT11 tag is ineffective in improving the solubility of taCA. In contrast, as previously shown, no precipitation was evident when assessing the NEXT-taCA, resulting in a visually clear enzyme solution (Figure 2b).

### 2.3. Improved Long-Term Thermal Stability of taCA by the Fusion of NEXT Tag

**Figure 3:**
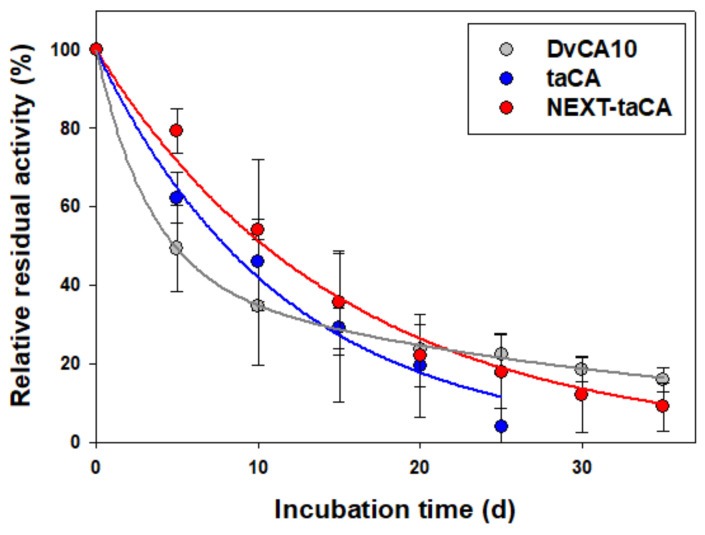
Time-course enzyme inactivation. Enzymes were incubated at 70 °C in 20 mM phosphate buffer (pH 7.5) supplemented with 300 mM NaCl, and the residual activities were measured by CO_2_ hydration assay. Solid lines represent the fitted regression curves. Error bars represent standard deviations from two independent experiments.

To evaluate the impact of the NEXT tag fusion on the long-term thermal stability of taCA, the taCA enzymes were incubated at 70 °C in a phosphate buffer supplemented with 300 mM NaCl for up to 35 days, and their residual activities were measured (Figure 3). DvCA10, an ultrastable CA engineered by directed evolution, was also tested under the same condition for comparison [28]. The two enzymes, taCA and NEXT-taCA, showed the general first-order inactivation kinetics that can be described as follows:

(1) $$\mathrm{Residual}\;\mathrm{activity}\left(\%\right)=e^{-kt}\times 100$$

where *k* is the inactivation rate constant, and t is the incubation time. In the case of DvCA10, the data were better fitted to the biphasic three-parameter kinetic model. It is known that enzyme inactivation kinetics can be described by the simple three-parameter model regardless of the underlying mechanism for the inactivation [29]. The three-parameter model can be expressed by the sum of two first-order equations as follows:

(2) $$\mathrm{Residual}\;\mathrm{activity}\left(\%\right)=\left(x_{1}e^{-k_{1}t}+x_{2}e^{-k_{2}t}\right)\times 100$$

where *x*_1_ and *x*_2_ are pre-exponential parameters; *x*_1_ + *x*_2_ = 1, *k*_1_ and *k*_2_ are apparent rate constants, and t is the incubation time. Considering only the apparent rate constant *k*_2_, DvCA10 was 3.2-fold more stable than taCA under the experimental condition (Table 1).

Remarkably, the fusion of the NEXT tag resulted in a 30% enhancement in the long-term thermal stability of taCA (Figure 3 and Table 1). This result seems contradictory to the previous study’s findings, where the thermal stability of NEXT-taCA was reported to be almost the same as that of the untagged taCA when their stability was assessed after heat exposure for 1 h at 90 °C [18]. This apparent discrepancy might arise from the different timescales for the inactivation kinetics and the aggregation kinetics of the soluble enzymes under exceptionally high-temperature conditions; the short-term exposure to 90 °C might induce rapid enzyme denaturation before enzyme aggregation significantly influences the overall enzyme stability, resulting in the seemingly similar stabilities between the NEXT-taCA and the untagged taCA. On the other hand, under the relatively mild condition of 70 °C, the inactivation and aggregation kinetics might have similar timescales. Since enzyme aggregation and precipitation contribute to a reduction in enzymatic activity, the precipitation of the untagged taCA before heat-induced denaturation could expedite the overall enzyme deactivation. These results underscore that the long-term thermal stability of taCA can benefit from the enhanced enzyme solubility conferred by the fusion of the NEXT tag.

### 2.4. Bioprospecting of Novel IDP-Based Solubility Enhancers

**Figure 4:**
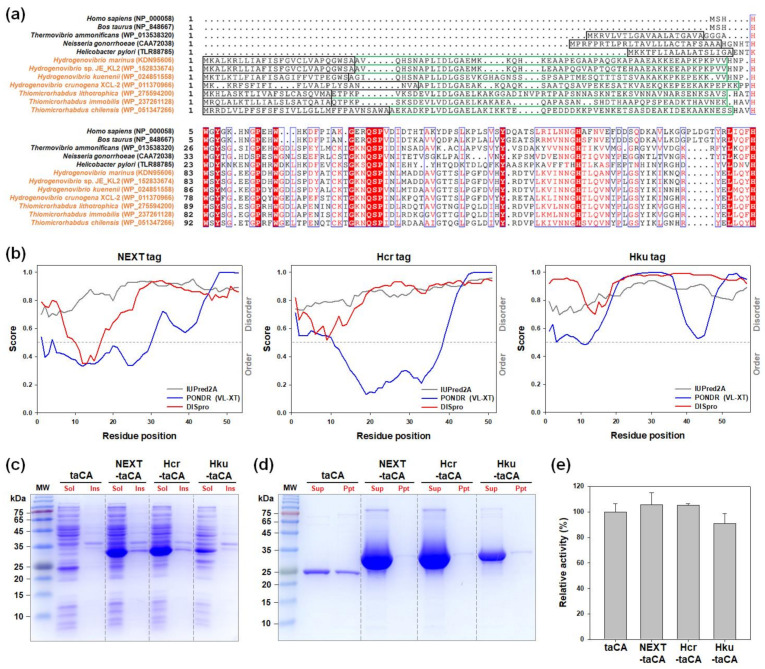
Bioprospecting of novel IDP-based solubility enhancers. (**a**) Multiple sequence alignment of α-type CAs. Only the N-terminal parts of the entire sequences are shown. The predicted signal sequences are boxed in black. The unique N-terminal extension sequences are enclosed in green boxes. Blue boxes indicate columns with strictly (red background) or 70% (red characters) conserved sequences across all the aligned protein sequences. (**b**) Intrinsic disorder propensities. Position-dependent prediction of disordered regions was performed using three different methods (IUPred2A, PONDR, and DISpro). The dashed line corresponds to the cutoff threshold score for the determination of the disordered region. (**c**) Expression analysis by SDS–PAGE followed by Coomassie blue staining. Lanes: MW, molecular mass marker; Sol, soluble fraction; Ins, insoluble fraction. (**d**) In vitro solubility of purified enzyme. After dialysis against 20 mM of sodium phosphate buffer (pH 7.5), protein precipitates were analyzed by SDS–PAGE followed by Coomassie blue staining. Lanes: MW, molecular mass marker; Sup, supernatant; Ppt, precipitate. (**e**) Activity of purified enzyme. The activities were measured by CO_2_ hydration assay and normalized to the activity of the untagged taCA. Error bars represent standard deviations from two independent experiments.

As previously mentioned, the NEXT tag originated from the N-terminal extension sequence of hmCA. Notably, genome sequencing has revealed that the presence of the unusual N-terminal extension is not exclusive to hmCA but is a shared characteristic among various α-CAs identified in widely distributed marine chemolithoautotrophic γ-*Proteobacteria* belonging to the genera *Hydrogenovibrio*, *Thiomicrorhabdus*, and *Thiomicrospira* [30]. These N-terminal sequences are unique in that they are absent in α-CAs from eukaryotes and other bacterial species (Figure 4a), and no sequence has been known with homology to them. Similar to the case of the NEXT tag, it was speculated that these unique N-terminal sequences may also be used as solubility-enhancing fusion tags.

To this end, the N-terminal extension sequences were selected from *H. crunogena* XCL-2 and *H. kuenenii* and were designated as Hcr tag and Hku tag, respectively. These were predicted as almost entirely IDP, along with the NEXT tag (Figure 4b). All other sequence properties, such as the sequence length, molecular mass, pI, and hydrophobicity, were similar (Table 2). When the novel IDP tags were fused to the taCA, the expression level of taCA was improved by both the Hcr and Hku tags. Notably, the performance of the Hcr tag was comparable to that of the NEXT tag (Figure 4c). The tagged taCA variants were purified along with the untagged taCA, and their in vitro solubility was examined. All of the tagged taCA enzymes exhibited no protein precipitation, while significant precipitation occurred with the untagged counterpart, demonstrating the effectiveness of the novel IDP-based solubility enhancers (Figure 4d). In addition, the activity changes of taCA caused by the fusion of the Hcr and Hku tags were marginal (Figure 4e), showing that these IDP-based fusion tags exert minimal effects on the taCA enzyme. These results suggest that the novel Hcr and Hku tags have the potential to be powerful solubility enhancers. Meanwhile, future analysis of the unique N-terminal extension sequences may reveal the sequence characteristics of IDPs that are essential for use as solubility-enhancing tags.

## 3. Materials and Methods

### 3.1. Strains and Construction of Expression Vectors

The strains, plasmids, and oligonucleotide primers used in this study are listed in Table 3. *E. coli* TOP10 (Thermo Fisher Scientific, Waltham, MA, USA) was used for the construction of plasmid vectors, and *E. coli* BL21(DE3) (Novagen, Madison, WI, USA) was used for recombinant protein expression. The cells were routinely cultivated in Luria–Bertani (LB) medium supplemented with appropriate antibiotics (50 μg/mL of ampicillin for recombinant strains or 10 μg/mL of streptomycin for wild-type *E. coli* TOP10) at 37 °C and 220 rpm in a shaking incubator (Jeiotech, Daejeon, Korea). The genes for NEXT tags with different linker lengths were amplified by polymerase chain reaction (PCR) using the primers listed in Table 3 and the previously constructed pET-NEXT-taCA [18] as the template. The PCR products were ligated into the pGEM-T Easy vector (Promega, Madison, WI, USA), and the insert sequences were confirmed by Sanger sequencing (Genotech, Daejeon, Korea). The genes were subcloned into pET-NEXT-taCA treated by *Nde*I and *Nco*I restriction enzymes by replacing the original NEXT tag sequence, resulting in pET-NEXT-taCA_no link_ and pET-NEXT-taCA_short link_. The DvCA10 gene [28] was codon-optimized, synthesized (Genscript, Piscataway, NJ, USA), and subcloned into pET-22b(+) vector (Novagen, Madison, WI, USA) using *Nde*I and *Xho*I sites, resulting in pET-DvCA10. The genes for Hcr tag and Hku tag were chemically synthesized (Genotech, Daejeon, Korea) and subcloned into pET-NEXT-taCA using *Nde*I and *Nco*I sites by replacing the NEXT tag sequence, resulting in pET-Hcr-taCA and pET-Hku-taCA. The vector pET-NT11-taCA was kindly gifted by Professor Seung Pil Pack (Korea University, Republic of Korea) [22]. The recombinant genes had a hexahistidine (His_6_)-tag encoding sequence at their 3′ terminus.

### 3.2. Purification of Recombinant Proteins

The recombinant protein was purified by immobilized metal affinity chromatography via His_6_-tag. After cell lysis, the soluble fraction was mixed with Ni^2+^-nitrilotriacetic acid agarose beads (Qiagen, Germantown, MD, USA), and the recombinant protein was purified according to the manufacturer’s instructions. The protein was eluted using elution buffer (50 mM of sodium phosphate, 300 mM of NaCl, and 250 mM of imidazole; pH 8.0). The eluate was thoroughly dialyzed against enzyme buffer (20 mM of sodium phosphate buffer and 300 mM of NaCl; pH 7.5) at 4 °C. After dialysis, any protein precipitates were removed by centrifugation at 10,000× *g* at 4 °C for 10 min. The supernatants were used for subsequent activity and stability tests.

### 3.3. In Vitro Solubility Test

The eluate was dialyzed against low-salt buffer (20 mM of sodium phosphate; pH 7.5) at 4 °C. After dialysis was completed, protein precipitates were separated by centrifugation at 10,000× *g* at 4 °C for 10 min. The precipitate fraction was resuspended in the same buffer and analyzed by SDS–PAGE along with the supernatant fraction.

### 3.4. Protein Analyses

For protein quantification, the purified protein was denatured in a denaturing buffer (6 M of guanidine hydrochloride GuHCl/20 mM of sodium phosphate buffer; pH 7.5), and the absorbance of the denatured protein was measured at 280 nm in a quartz crystal cuvette (Hellma Analytics, Müllheim, Germany). The protein concentration was determined using the measured absorbance and the calculated molar extinction coefficient at 280 nm [31]. Protein samples were separated by SDS–PAGE on 15% PAGE gel and visualized by Coomassie blue R-250 (Bio-Rad, Hercules, CA, USA) staining.

### 3.5. CO_2_ Hydration Assay

Enzyme activity was measured via a colorimetric CO_2_ hydration assay modified from the Wilbur–Anderson method [32,33]. The assay was performed at 0 °C inside the spectrophotometer equipped with a temperature-controllable cell holder. Briefly, 10 μL of the sample was added to a disposable cuvette containing 600 μL of 20 mM Tris buffer (pH 8.3) supplemented with 100 μM of phenol red (Sigma-Aldrich, St. Louis, MO, USA). The CO_2_ hydration reaction was initiated by adding 400 μL of CO_2_-saturated water prepared in ice-cold water. The absorbance change was monitored at 570 nm, and the time (*t*) required for the pH drop from 7.5 to 6.5 was obtained. The time (*t*_0_) for the uncatalyzed reaction was also measured by adding the corresponding blank buffer. The Wilbur–Anderson unit (WAU) was calculated as (*t*_0_ − *t*)/*t*.

### 3.6. Thermal Inactivation Test

The concentration of purified enzyme was adjusted to 10 μM. The enzymes (taCA, NEXT-taCA, and DvCA10) were incubated at 70 °C in a water bath (Jeiotech, Daejeon, Korea). They were then stored at 4 °C until their activities were measured. The CO_2_ hydration activities of the incubated samples were measured and compared with the activity of the nonincubated sample. The relative residual activity was calculated as follows:

(3) $$\mathrm{Relative}\;\mathrm{residual}\;\mathrm{activity}\left(\%\right)=\left(\frac{\mathrm{Activity}\;\mathrm{of}\;\mathrm{heat}-\mathrm{treated}\;\mathrm{sample}}{\mathrm{Activity}\;\mathrm{of}\;\mathrm{untreated}\;\mathrm{sample}}\right)\times 100$$

### 3.7. In Silico Analyses

Densitometric analysis of the protein band on the gel was performed using ImageJ 1.50i [34]. Protein parameters, including molar extinction coefficient, molecular mass, number of charged amino acids, and isoelectric point (pI), were calculated by ProtParam (http://web.expasy.org/protparam/ (accessed on 3 December 2023)) [35]. The Kyte–Doolittle hydropathy index was calculated by ProtScale (https://web.expasy.org/protscale (accessed on 3 December 2023)) using a window size of 5, and the values were averaged to obtain a mean hydropathy index [35,36]. Signal peptide cleavage sites were predicted by the SignalP 6.0 server (https://services.healthtech.dtu.dk/services/SignalP-6.0/ (accessed on 2 December 2023)) [37]. Multiple sequence alignment was performed using ClustalX 2.0 [38], and the result was visualized by ESPript 3.0 (https://espript.ibcp.fr/ESPript/ESPript/ (accessed on 2 December 2023)) [39]. Disordered propensity was predicted by IUPred2A (https://iupred2a.elte.hu (accessed on 1 December 2023)) [40], PONDR (http://www.pondr.com (accessed on 1 December 2023)) [41], and DISpro (http://scratch.proteomics.ics.uci.edu (accessed on 1 December 2023)) [42].

## 4. Conclusions

The length of the linker influenced the expression level but did not appear to be a critical factor for improving the solubility of taCA. The NEXT tag proved effective in improving both the expression and solubility of taCA, whereas the NT11 improved only the expression level. The enhanced solubility by the fusion of the NEXT tag contributed to the improved thermostability of taCA, presumably by limiting enzyme aggregation and thereby alleviating activity loss. Two novel solubility-enhancing tags were discovered and examined for their impact on the improved expression and solubility of taCA. This study sheds light on the diverse aspects of the NEXT tag and its related sequences, providing valuable tools and insights for the recombinant expression of enzymes.

## Figures and Tables

**Table 1:** Kinetic values for thermal inactivation of enzyme at 70 °C.

	Kinetic Mode	Pre-Exponential Parameters	Inactivation Rate Constants (Half-Life in Day)
DvCA10	Biphasic	*x*_1_: 0.58	*k*_1_: 0.3064 d^−1^ (2.3 d)
		*x*_2_: 0.42	*k*_2_: 0.0272 d^−1^ (25.5 d)
taCA	Monophasic	1	*k*: 0.0867 d^−1^ (8.0 d)
NEXT-taCA	Monophasic	1	*k*: 0.0667 d^−1^ (10.4 d)

**Table 2:** Sequence properties of IDP-based solubility-enhancing tags used in this study.

Fusion Tag	Amino Acid Length	Molecular Mass (kDa)	pI	Charged Amino Acids	Net Charge	Mean Hydropathy
NEXT	53	5.5	8.1	28%	+1	0.397
Hcr	51	5.5	9.0	39%	+2	0.367
Hku	57	5.8	9.2	21%	+2	0.406

**Table 3:** Strains, plasmids, and oligonucleotide primers used in this study.

Strains, Plasmids, or Primers	Genotypes, Relevant Characteristics, or Sequences	Source or References
Strains		
*E. coli* TOP10	F^−^ *mcrA* Δ*(mrr-hsdRMS-mcrBC)* Φ80*lacZ*ΔM15 Δ*lacX*74 *recA*1 *araD*139 Δ*(ara-leu)*7697 *galU galK rpsL*(Str^r^) *endA*1 *nupG*	Thermo Fisher Scientific, Waltham, MA, USA
*E. coli* BL21(DE3)	F^−^ *ompT hsdS*_B_(r_B_^−^ m_B_^−^) *gal dcm lon* λ(DE3), carrying T7 RNA polymerase gene	Novagen, Madison, WI, USA
Plasmids		
pGEM-T Easy	pUC *ori*, Amp^r^, TA cloning vector,	Promega, Madison, WI, USA
pET-22b(+)	T7*lac* promoter, pBR322 *ori*, Amp^r^, parental expression vector harboring PelB signal sequence	Novagen, Madison, WI, USA
pET-taCA	Expression plasmid carrying taCA gene	[18]
pET-NEXT-taCA	Expression plasmid carrying NEXT-tagged taCA gene with the (GGGGS)_2_ linker	[18]
pET-NEXT-taCA_no link_	Expression plasmid carrying NEXT-tagged taCA gene without linker	This study
pET-NEXT-taCA_short link_	Expression plasmid carrying NEXT-tagged taCA gene with the GGGGS linker	This study
pET-NT11-taCA	Expression plasmid carrying NT11-tagged taCA gene	[22]
pET-DvCA10	Expression plasmid carrying DvCA10 gene	This study
pET-Hcr-taCA	Expression plasmid carrying Hcr-tagged taCA gene with the (GGGGS)_2_ linker	This study
pET-Hku-taCA	Expression plasmid carrying Hku-tagged taCA gene with the (GGGGS)_2_ linker	This study
Primers ^a^		
NEXT-Forward	CATATGGCTGTTCAACATAGCAATGCCCC	[18]
NEXT-no link-Reverse	CCATGGCCACAACGGGTTTTGGTTTAG	This study
NEXT-short link-Reverse	CCATGGAGCCTCCACCGCCCACAACGGGTTTTGGTTTAG	This study

^a^ Restriction sites are underlined.

## Data Availability

Data will be made available on request.
